# A Cross-Sectional Study to Analyze the Correlation Between Alexithymia and Dental Neglect in Persons Pursuing Dental Care

**DOI:** 10.7759/cureus.43341

**Published:** 2023-08-11

**Authors:** Sneha Suresh, Ranjita Singh Baghel, Abhishek Gautam, Anant Kumar, Deep Sundar, Suman Kriti

**Affiliations:** 1 Department of Periodontology, Buddha Dental College and Hospital, Patna, IND; 2 Department of Periodontics, Index Institute of Dental Sciences, Indore, IND; 3 Department of Periodontology and Implantology, Government Dental College and Hospital, Nalanda, IND; 4 Department of Public Health Dentistry, Ambika Dental Care, Patna, IND; 5 Department of Public Health Dentistry, Sanjeevani Dental Hospital, Patna, IND; 6 Department of Public Health Dentistry, Buddha Institute of Dental Science and Hospital, Patna, IND

**Keywords:** tas-20, dental patients, dental neglect scale, dental neglect, dental care, alexithymia

## Abstract

Background

Alexithymia is a personality trait involving difficulties in emotional regulation (difficulties in identifying feelings, difficulties in describing feelings, and externally oriented thinking). It has a negative impact on health as it evokes poor personal hygiene, poor nutrition, and unhealthy behaviors in affected subjects. Identifying alexithymia in the dental setup is vital as it can compromise the patient-dentist relationship, especially in subjects neglecting oral hygiene.

Aims

The present study aimed to establish an association between alexithymia and dental neglect among adult subjects seeking dental care by using Dental Neglect Scale (DNS), and alexithymia was assessed on the 20-item Toronto Alexithymia Scale (TAS-20).

Methods

The present cross-sectional survey study included adult subjects of age 20 years or more. For all included participants, a structured questionnaire was given to assess dental neglect on demographic profile, six items of the DNS, and alexithymia was assessed on the 20-item TAS-20. The collected data were analyzed using a Chi-square test keeping significance at the p-value of <0.05.

Results

In 534 adult subjects, females had high scores for both TAS-20 and DNS along with their related factors. With higher education and increasing age, a significant increase in the mean TAS-20 scores and mean DNS scores was seen in the study participants (high mean DNS scores in females (19.55±3.98) compared to male subjects 19.36±4.34). TAS-20 scores were higher in females (59.31±10.78), factor 1 (DIF) (19.54±5.54), factor 2 (DDF) (15.46±4.05), and factor 3 (EOT) (24.34±4.64).

Conclusion

The present study, considering its limitations, concludes that there is no association between dental neglect and alexithymia in adult subjects seeking dental care. However, higher DNS and TAS-20 scores are seen in females showing them have difficult descriptions and identification of feelings in dental set-up increasing dental neglect among them.

## Introduction

Alexithymia is a condition that links health to emotions and is considered a stable personality trait (i.e., people are supposedly consistent in their motivations and actions). Alexithymia is explained as a multifaceted construct posing difficulties in the affected subjects concerning stimulus-bound cognitive style, externally oriented cognitive style, difficulty describing feelings (DDF) to others, emotional arousal-like sensations, difficulty distinguishing different feelings, and identifying subjective feelings. These conditions put social, emotional, and physical burdens on the affected subjects as suggested by Mukesh in 2021 [1].

Alexithymia also affects the overall health of the affected individuals by awakening the unhealthy behavioral patterns in these subjects. Alexithymia also makes communication between clinicians and patients difficult along with failure to recognize or experience different adaptive feelings including self-pride, guilt, and/or fear [2].

Recent/this study/current study work assessed the association between alexithymia and its impact on oral health. Oral health has a vital role and contribution to the overall well-being and health of humans. Also, oral disorders are being considered lifestyle and diet-related diseases posing a high burden on the financial status of the affected subject as suggested by Ismail et al. in 2023 [2].

Along with being financial impact, oral diseases also pose a social and personal burden on the subjects due to the pain associated with the oral conditions. Previous research work also assessed dental neglect (DN) in the American setup utilizing a self-reported scale comprising nine items assessing the three aspects of general neglect, service use, and self-care.

According to this study, DN may be evaluated objectively, and there is a relationship between DN and dental health that can be evaluated as mentioned by Rath et al. [3]. DN is defined as attitudes and behaviors that can have a degrading effect and result in the overall health of the affected subjects as mentioned by Rath et al. [3]. At every point in one's life, DN is noticed and reported and is associated with different etiologic factors at different points in time. Hence, identifying alexithymia in subjects seeking dental care is vital as it has a negative impact on the patient-dentist relationship, particularly, in subjects neglecting their dental hygiene [4].

Alexithymia and oral health-related concerns have been linked by various previous studies, which were conducted in different years. One such study in 2009 reported that few subjects having dental anxiety can have difficulty describing and identifying their feeling and fears to their treating dentists as assessed in Finnish adult subjects seeking dental care. Another literature work concluded that dental fear is much higher in alexithymic subjects compared to non-alexithymic subjects. Poor oral health-related quality of life is also seen in subjects with alexithymia having emotional dysregulation. Recent studies showed that in subjects with alexithymia, low oral health literacy is noted, as assessed in subjects visiting dental care settings. This was concluded from the study conducted on the subjects from Norway [5].

There is not yet concrete evidence linking alexithymia to DN. It has not been proven to be a screening tool or risk factor for issues connected to poor oral health. There are few studies analyzing the relationship between alexithymia and DN in the Indian dentistry system. The purpose of the current study was to determine if DN among adult patients seeking dental care is associated with alexithymia.

## Materials and methods

The present study was done to evaluate the association between alexithymia and DN among adult subjects seeking dental care. The study was conducted at Buddha Institute of Dental Science and Hospital, Patna, Bihar (BIDSP/151/2023) after clearance was obtained from the Institutional Ethical Committee. The participants were recruited from the Outpatient Department of the Institute. Patients were randomly selected from the routine dental Outpatient Department. Only subjects with age 20 years-68 years were only screened to be included in the study. Informed consent for study participation was taken from all participants in both verbal and written format.

For the present study, a total sample size of 534 participants from both genders was 48.12% (n=257) males and 51.87% (n=277) females. The subjects were aged from 20 years to 68 years with a mean age of 35.42±10.38 years. After the study subjects were recruited, they were given a self-administered, performed, and structured questionnaire given to each participant in both Hindi and English for better understanding. This questionnaire was used as a survey tool and comprised three parts.

The first part of the questionnaire assessed the demographics of the participants including reason to visit the dental facility, previous dental visits, education, gender, and age of the participants. The second part was to evaluate DN using the DN Scale (DNS) by Thomson et al. [6] in 1996 with six items. These items assessed dental behavior with responses ranging from strongly agree allotted a score of 1 to strongly agree with a score of 5 rated on a Likert scale of 5 points. A negative record was done for items 3, 4, and 6, which were scored in reverse to make questions aggregate and balance in a negative/positive direction. The scores given were in the range of 6-30 where 6 showed the least DN and 30 showed most DN.

The third part of the survey tool assessed alexithymia on a scale comprising 20 items Toronto Alexithymia Scale (TAS-20) described by Bagby [7] in 1994. The 20 items of the TAS-20 scale were divided into three parameters where seven items were dedicated to difficulty identifying feelings (DIF) given scores of 1, 3, 6, 7, 9, 13, and 14, and five items to DDF given scores of 2, 4, 11, 12, and 17, and 8 items to externally oriented thinking (EOT) assigned the scores of 5, 8, 10, 15, 16, 18, 19, and 20. All 20 items were given a score of 1 showing strongly disagreeing and the score of 5 as strongly agree. The total scores were from 20 to 100.

The negatively recorded five items were 4, 5, 10, 18, and 19. Alexithymia was considered for a score of 61 or higher. The grading was done as non-alexithymia for a score of <51, intermediate for 52-60, and alexithymia for scores of ≥61.

Categories of five responses were made owing to the complex analysis including agree (strongly agree and moderately agree), neither disagree nor agree and disagree (moderately disagree and strongly disagree). The collected data were statistically evaluated using SPSS Version 22.0 (IBM Corp., Armonk, NY, USA) software using the Chi-square test for alexithymia and DN association, Mann-Whitney U-test and t-test for comparison of demographics to TAS-20 mean scores and DNS. The significance was considered for a p-value of <0.05. Also, the reliability and validity of the questionnaire were assessed.

## Results

The present study assessed 534 adult subjects seeking dental care with 48.12% (n=257) males and 51.87% (n=277) females. The subjects were aged from 20 years to 68 years with a mean age of 35.42±10.38 years. The majority of the study subjects were in the age range of 21-40 years with 72.09% (n=385) subjects followed by 22.47% (n=120) subjects in 41-60 years age, 3.55% (n=19) subjects in >60 years, and 1.87% (n=10) subjects of 20 years of age. The majority of the subjects had education of higher than high school in 21.34% (n=114) subjects followed by high school in 21.34% (n=114) participants, primary education in 10.48% (n=56) subjects, and 23.97% (n=128) subjects were uneducated. For the majority of the subjects, no previous dental visit was there as seen in 60.11% (n=321) subjects followed by <12 months in 16.85% (n=90) subjects. In subjects, the most common reason for last dental visit was tooth pain reported by 36.32% (n=194) subjects followed by general check-up in 27.71% (n=148) subjects, oral prophylaxis in 21.72% (n=116) subjects, tooth caries in 8.42% (n=45) subjects, orthodontic reason in 3.74% (n=20) subjects, and prosthodontic reasons in 2.05% (n=11) subjects respectively as shown in Table 1.

**Table 1:** Comparison of TAS-20 scores to different study parameters TAS-20: Toronto Alexithymia Scale, DIF: Difficulty Identifying Feelings, DDF: Difficulty Describing Feelings, EOT: Externally Oriented Thinking

Parameters	Subgroup	TAS-20	Factor 1 (DIF)	Factor 2 (DDF)	Factor 3 (EOT)
Age (years)	20	61.52±11.47	20.87±6.29	16.27±3.24	24.6±4.52
	21-40	58.87±11.07	19.38±5.70	15.13±4.15	24.5±3.49
	41-60	59.97±11.38	19.50±5.75	16.14±3.80	24.35±3.6
	>60	53.48±15.02	16.53±7.04	14.61±5.41	22.37±4.05
	P-value	0.007	0.05	0.16	0.05
Dental visit reason	Prosthodontics	64.88±9.73	22.59±3.94	17.78±3.73	24.54±3.3
	Orthodontics	58.47±11.28	20.15±7.34	14.54±4.20	23.73±2.99
	Tooth caries	59.82±9.37	19.6±4.14	15.52±3.75	24.3±3.36
	Tooth Pain	58.54±12.36	19.08±6.39	15.27±4.35	24.25±4.45
	Oral prophylaxis	58.40±11.17	18.79±5.04	15.6±4.23	24.4±3.73
	General check-up	59.39±10.5	19.65±5.75	15.33±3.85	24.47±3.54
	P-value	0.001	0.26	0.47	0.002
Gender	Males	58.54±11.97	19.3±5.9	15.17±4.29	24.24±4.19
	Females	59.31±10.78	19.54±5.54	15.46±4.05	24.34±4.64
	P-value	0.25	0.21	0.05	0.6
Last dental visit	None	59.94±10.41	19.88±5.65	15.67±3.83	24.61±3.69
	<12 months	59.22±11.97	19.55±5.7	15.63±4.13	24.21±4.47
	1-2 years	56.62±12.3	18.38±5.06	14.21±4.52	23.75±3.76
	2-5 years	56.51±12.6	17.77±5.69	14.69±4.89	23.79±4.03
	P-value	0.001	0.001	0.001	0.004
Educational status	Uneducated	61.21±9.79	20.50±5.25	16.20±3.45	24.53±3.79
	Primary	60.35±10.84	20.06±5.53	15.85±4.05	24.46±3.67
	High school	59.75±10.06	19.91±5.42	15.65±3.92	24.4±3.1
	Higher	57.18±12.45	18.5±6.11	14.69±4.45	24.16±4.28
	P-value	0.001	0.001	0.001	0.27
	Total	58.98±11.35	19.34±5.79	15.36±4.13	24.31±3.89

Most of the study participants answered DNS 1, 2, 4, and 6 as agreed when comparing the various characteristics on the DNS, which has six items. Many survey participants chose the disagree response to DNS 3, which meant that they had put off getting the dental treatment they needed. Additionally, a sizable majority of participants said they disagreed with the statement in DNS 5 that “I control snacking between meals as I should do.” Regarding age, mean DNS was substantially greater at age >60 years, 21.64±3.69, which fell to 18.31±3.86 at age 41-60, 19.72±4.23 at age 21-40, and 19.23±2.86 at age 20 with a p-value of 0.001. The patients receiving orthodontic treatment had the highest mean DNS scores, 21.47±2.86, followed by those receiving a general checkup, 19.46±4.4, 19.43±4.46, 19.39±3.6, 19.27±3.91, and 16.93±3.36 for tooth pain, oral prophylaxis, tooth caries, and prosthodontics in that order, respectively.

With a p-value of 0.001, this was statistically very significant. Subjects with a dental appointment during the previous 12 months had substantially higher DNS mean scores than those with visits within the previous two years, three years, or none at all (p 0.001). Mean DNS scores climbed in subjects with high school and higher education, with corresponding mean DNS values of 19.31±4.33 and 20.46±3.95; these scores decreased in subjects with no education and subjects with elementary education, with 18.14±4.06 and 18.69±3.77 and 18.31±4.30 and 18.69±3.77, respectively. With a p-value of 0.45, this difference was, nevertheless, statistically insignificant (Table 2).

**Table 2:** Comparison of different study parameters to Dental Neglect Scale (DNS)

Parameters	Subgroup	DNS (Mean ± S. D)
Age (years)	20	19.23±2.86
	21-40	19.72±4.23
	41-60	18.31±3.86
	>60	21.64±3.69
	P-value	0.001
Gender	Males	19.36±4.34
	Females	19.55±3.98
	P-value	0.001
Dental visit reason	Prosthodontics	16.93±3.36
	Orthodontics	21.47±2.86
	Tooth caries	19.27±3.91
	Tooth Pain	19.43±4.46
	Oral prophylaxis	19.39±3.6
	General check-up	19.46±4.4
	P-value	0.001
Last dental visit	None	18.85±4.24
	<12 months	20.52±4.25
	1-2 years	20.42±19.45
	2-5 years	19.73±3.66
	P-value	0.001
Educational status	Uneducated	18.14±4.06
	Primary	18.69±3.77
	High school	19.31±4.33
	Higher	20.46±3.95
	P-value	0.45
	Total	19.45±4.18

The majority of participants agreed with TAS-17, which means “It is difficult for me to reveal my innermost feelings, even to close friends,” with regards to the TAS-20 scores. The subjects disagreed on the following 10 TAS-20 items: TAS 3, 4, 6, 8, 9, 10, 12, 13, 16, and 19. Participants chose neither disagree nor agree for TASs 18 and 7. For the TAS-20, mean values for factors 1 DDF and 3 EOT were greater for the age range of 20 years, with respective values of 20.87±6.29 and 24.6±4.52. As people aged, these mean values for factors 1 and 3 decreased considerably, with a p-value of 0.05 for each. The age difference for factor 2, DDF, however, was statistically insignificant with a p-value of 0.16 (Table 3).

**Table 3:** Comparison of TAS-20 scores to different study parameters

Parameters	Subgroup	TAS-20	Factor 1 (DIF)	Factor 2 (DDF)	Factor 3 (EOT)
Age (years)	20	61.52±11.47	20.87±6.29	16.27±3.24	24.6±4.52
	21-40	58.87±11.07	19.38±5.70	15.13±4.15	24.5±3.49
	41-60	59.97±11.38	19.50±5.75	16.14±3.80	24.35±3.6
	>60	53.48±15.02	16.53±7.04	14.61±5.41	22.37±4.05
	P-value	0.007	0.05	0.16	0.05
Dental visit reason	Prosthodontics	64.88±9.73	22.59±3.94	17.78±3.73	24.54±3.3
	Orthodontics	58.47±11.28	20.15±7.34	14.54±4.20	23.73±2.99
	Tooth caries	59.82±9.37	19.6±4.14	15.52±3.75	24.3±3.36
	Tooth Pain	58.54±12.36	19.08±6.39	15.27±4.35	24.25±4.45
	Oral prophylaxis	58.40±11.17	18.79±5.04	15.6±4.23	24.4±3.73
	General check-up	59.39±10.5	19.65±5.75	15.33±3.85	24.47±3.54
	P-value	0.001	0.26	0.47	0.002
Gender	Males	58.54±11.97	19.3±5.9	15.17±4.29	24.24±4.19
	Females	59.31±10.78	19.54±5.54	15.46±4.05	24.34±4.64
	P-value	0.25	0.21	0.05	0.6
Last dental visit	None	59.94±10.41	19.88±5.65	15.67±3.83	24.61±3.69
	<12 months	59.22±11.97	19.55±5.7	15.63±4.13	24.21±4.47
	1-2 years	56.62±12.3	18.38±5.06	14.21±4.52	23.75±3.76
	2-5 years	56.51±12.6	17.77±5.69	14.69±4.89	23.79±4.03
	P-value	0.001	0.001	0.001	0.004
Educational status	Uneducated	61.21±9.79	20.50±5.25	16.20±3.45	24.53±3.79
	Primary	60.35±10.84	20.06±5.53	15.85±4.05	24.46±3.67
	High school	59.75±10.06	19.91±5.42	15.65±3.92	24.4±3.1
	Higher	57.18±12.45	18.5±6.11	14.69±4.45	24.16±4.28
	P-value	0.001	0.001	0.001	0.27
	Total	58.98±11.35	19.34±5.79	15.36±4.13	24.31±3.89

Prosthodontics received the highest overall TAS-20 scores for the reason for the dental visit, followed by dental caries, tooth discomfort, orthodontics, and oral prophylaxis, while general check-up-related visits had the lowest scores (p=0.001). With corresponding p-values of 0.26 and 0.47, factor 1, DIF, and factor 2 (DDF) saw a non-significant difference for various dental visit causes. But with p=0.002, results for factor 3, EOT, were statistically significant. Overall, TAS-20 scores were higher in subjects who had never visited a dentist. However, factor 1 (DIF), factor 2 (DDF), and factor 3 (EOT) consistently decreased to 12 months, one to two years, and two to five years significantly for over TAS-20, factor 1 (DIF), factor 2 (DDF), and factor 3 (EOT), with respective p-values of 0.001, 0.001, 0.001, and 0.004, respectively. With relative p-values of 0.001, 0.001, and 0.001, similar findings were found for educational status for TAS-20 scores, factor 1 (DIF), and factor 2 (DDF). With a p=0.27 statistically non-significant value, factor 3, EOT, was the exception (Table 3).

## Discussion

DN describes the attitudes and behaviors in an individual leading to non-judgmental and inadequate evaluation and value of oral health which has been established as a predictive factor for poor oral health among adults as well as pediatric subjects. This can be assessed with various factors including the number of teeth lost, toothache, and indices of caries and plaque. Sometimes, the subjects with DN can have difficulty in understanding and communicating their information, and fears and identifying and telling their feelings to the treating dentist. Alexithymia is one such personality condition where the affected subject has trouble expressing, describing, and identifying their feelings. Alexithymia originally belonged to psychosomatic medicine denoting no words to describe feelings. Identification of alexithymia in subjects with DN is considered vital as it can affect the relationship between patient and dentist, affecting the ultimate treatment outcomes as reported by Mattila et al. [8] in 2012. Hence, the present study was done to assess an association between alexithymia and DN among adult subjects seeking dental care.

The present study utilized DNS for assessing DN. The Dental Indifference Scale (DIS) can also be used to identify DN. However, the DIS was described to be difficult data manipulation, time-consuming, difficult to derive the scores, and difficult to use Jamieson and Thomson [9] in 2002. On the contrary, DNS has various advantages that can be utilized in a dental setting being easy to use and allowing identification of individuals or groups that can take advantage of health promotion.

Similarly, alexithymia can be assessed using various questionnaires including Online Alexithymia Questionnaire, 40-item Bermond-Vorst Alexithymia Questionnaire (BVAQ-40), and TAS-20. However, BVAQ-40 has shown to be inconsistent in a few dimensions as reported by Morera et al. [10] in 2005 considering TAS-20 as a popular and reliable assessment tool, which was also utilized in the present study.

In the present study, there were 51.87% (n=277) females which is consistent with the Finnish study by Viinikangas et al. [11] in 2009 where the authors assessed the study population having 62.6% females and the Norwegian study by Stein et al. in 2015 where authors assessed a population with the female predominance of 56%. This female predominance in these studies can be attributed to the fact that esthetic concerns are high in female subjects with more concern about oral hygiene. Another reason for female predominance was reported by Shaygali et al. [12] who reported that dental institutes in India are open during the daytime making it difficult for male subjects to visit them during working hours.

The present study results showed high mean DNS scores in females, 19.55±3.98 compared to male subjects (19.36±4.34). These results were contradictory to the results of McGrath et al. [13] in 2007 and Thomas and Locker [14] in 2000 where mean DNS scores were higher in males compared to females conducted in Dunedin and Hong Kong Chinese subjects, respectively. These differences can be females having more fear and anxiety about dental treatment. Also, in the present study TAS-20 scores were higher in females for TAS-20 (59.31±10.78), factor 1 (DIF) (19.54±5.54), factor 2 (DDF) (15.46±4.05), and factor 3 (EOT) (24.34±4.64), whereas higher TAS-20 scores for males were reported by Parker et al. [15] in 2003, which can be due to more emotion and feelings sharing in females, especially to close persons. 

In the present study, TAS-20, factors 1, 2, and 3 decreased with increasing age with respective p-values of 0.007, 0.05, 0.16, and 0.05, However, this was in contrast to the results of Onor et al. [16] in 2010 higher mean TAS-20 scores were fund in adults compared to child subjects. This can be due to more awareness in adults about emotions and health. Also, higher mean DNS scores were seen for subjects with higher education (20.46±3.95). This was similar to the study of Sarkar et al. [17] in 2015 where a high DNS of 19.77±3.94 was reported in Pharmacy students warranting counselling and awareness programs.

The study's limitations were a small sample size and geographic bias. Socioeconomic status should also be taken into account. The absence of a psychologic status assessment and results based solely on individuals' self-declarations were a couple of the study's drawbacks.

## Conclusions

The present study concludes no association exists between DN and alexithymia in adult subjects seeking dental care. However, higher DNS and TAS-20 scores are seen in females showing them have difficult descriptions and identification of feelings in dental set-up increasing DN among them. Oral hygiene habits and health awareness campaigns are necessary for better results. Additional studies with detailed psychological evaluations can help to reach a definitive conclusion.
